# Prevalence of Anxiety and Depression Among Women With Polycystic Ovarian Syndrome: A Cross-Sectional Study From a Tertiary Care Hospital of Islamabad, Pakistan

**DOI:** 10.7759/cureus.52540

**Published:** 2024-01-19

**Authors:** Arham Yahya Rizwan Khan, Muhammad Areeb Abdullah, Rumaan Gul, Haider Raza Bhutta, Maryam Imran, Syeda Batool Mazhar, Nabia Tariq

**Affiliations:** 1 Medicine, Shifa College of Medicine, Shifa Tameer-e-Millat University, Islamabad, PAK; 2 Obstetrics and Gynecology, Pakistan Institute of Medical Sciences, Islamabad, PAK; 3 Obstetrics and Gynecology, Maroof International Hospital, Islamabad, PAK

**Keywords:** mental health, prevalence, depression, anxiety, polycystic ovary syndrome

## Abstract

Background

Depression and anxiety are common psychological conditions associated with polycystic ovarian syndrome (PCOS). It is important to understand the role of various demographic and socio-economic factors that contribute to the development of these psychological conditions.

Objectives

The aims of this study were to determine the prevalence of anxiety and depression in women with PCOS and to find the association of various demographic and socio-economic factors with anxiety and depression.

Methods

This was a single-center cross-sectional study conducted at a tertiary care hospital in Islamabad, Pakistan, from May 2021 to August 2022. All female patients, aged 18 to 40 years and diagnosed with PCOS, who presented to the department of Gynecology during the study period were eligible to be enrolled in the study. The Hospital Anxiety and Depression scale (HADS) was used to determine the level of anxiety and depression in the participants. HADS comprises 14 items scored on a Likert scale ranging from 0 to 3. Seven items correspond to depression and anxiety each. The scores range from 0 to 21 for both domains. A score of 7 or less was considered normal, 8-10 as borderline, and 11 or above as abnormal for both anxiety and depression. Data were analyzed using IBM SPSS Statistics for Windows, Version 26.0 (IBM Corp., Armonk, NY, USA).

Results

A total of 74 patients with PCOS were included in the study. The mean age of all the participants was 26.8 ± 5.2 and the mean body mass index (BMI) was 28.7 ± 5.4. The presence of PCOS-related symptoms was observed in all 74 cases. Menstrual cycle abnormalities were the most common symptom, which was present in 57 (77.0%) cases, followed by weight gain, which was present in 50 (67.6%) cases, and hirsutism, which was present in 41 (55.4%) cases. Diabetes mellitus and hypertension were present only in three (4.1%) and two (2.7%) cases, respectively, and positive family history of depression and/or anxiety was reported by 20 (27%) cases. The mean HAD score was 7 ± 3.8 for depression and 8 ± 3.7 for anxiety. Depression was diagnosed in 13 (17.6%) cases, and anxiety was diagnosed in 15 (20.3%) cases. Depression was found to be significantly associated with BMI (p = 0.015), level of education (p = 0.033), and monthly household income (p = 0.004). Anxiety was found to be associated with employment status (p = 0.009) and current pregnancy (p = 0.007). Rest of the factors such as age, marital status, ethnicity, menstrual irregularities, comorbidities such as diabetes mellitus and hypertension, and a family history of PCOS, anxiety, or depression did not show statistically significant association with either anxiety or depression (p < 0.05).

Conclusion

Anxiety and depression are common in patients with PCOS. These psychological conditions are associated with various demographic and socio-economic factors such as BMI, level of education, monthly household income, employment status, and pregnancy. It is recommended to involve a multidisciplinary team while managing patients with PCOS to timely identify and treat these psychological conditions in these patients.

## Introduction

Polycystic ovarian syndrome (PCOS), also called Stein-Leventhal syndrome, is an endocrine disorder that primarily affects the female reproductive system. It is a heterogeneous condition that affects approximately one in 10 women in their reproductive age [1]. Hormonal imbalances, such as elevated serum luteinizing hormone (LH) levels, increased levels of androgens as compared to estrogens, and raised insulin resistance are commonly seen in this disorder. The hormonal imbalance results in various clinical manifestations such as oligomenorrhea or amenorrhea, hirsutism, diabetes, obesity, infertility, and acne [2]. The disease is diagnosed using the Rotterdam criteria, according to which at least two of the following parameters must be present: polycystic ovaries on ultrasound, oligo-anovulation, and hyperandrogenism [3]. Once diagnosed, the condition is managed with a combination of lifestyle modifications (healthy diet, exercise), hormonal pills, anti-androgen therapy, metformin, ovulation induction, and, if needed, artificial reproductive techniques [4].

An integral component of the biopsychosocial model of healthcare includes good psychiatric health, and PCOS is no exception to this. The complications of PCOS can have multiple psychological effects on women. Research has shown that the prevalence of depression in women with PCOS is higher (28-64%) as compared to the general population (8%) [5-7]. The same is also true when we compare the prevalence of anxiety in PCOS patients (34-57%) to the general population (8%) [7-9]. These mental conditions adversely affect the quality of life of these patients and it is due to these psychosocial manifestations that women with PCOS are at an increased risk of social phobia and suicide attempts [9].

The prevalence of PCOS in the South Asian population including Pakistan is a matter of grave concern. Studies have reported a prevalence as high as 52% in Pakistani population as compared to 20-25% in Western populations [10]. The prevalence of depression and anxiety in the Pakistani PCOS patients also appears to be higher than other regions of the world. Local studies have reported an astonishingly high prevalence of depression (56.9%) and anxiety (61.8%) [11]. Additionally, the literature is evident that the awareness regarding PCOS and its psychological manifestations is less than 35% [12]. It, therefore, becomes imperative to assess the current prevalence and the factors that play a role in the development of these psychological manifestations. Knowledge of current prevalence and the role of various contributing factors will create awareness among the healthcare professionals and highlight the need for early screening and diagnosis of these psychological manifestations. This can serve to ensure enhanced quality of life of patients with PCOS, which is the ultimate goal of this research. This study was, therefore, conducted with the aims to determine the prevalence of anxiety and depression in women with PCOS presenting to a tertiary care hospital in Islamabad, Pakistan, and to find the association of various demographic and socio-economic factors with the development of anxiety and depression in women with PCOS.

## Materials and methods

Study overview

This was a single-center cross-sectional study conducted at a tertiary care hospital in Islamabad, Pakistan, from May 2021 to August 2022.

Ethical consideration

The approval for the study was taken from the Hospital Ethics Committee of Pakistan Institute of Medical Sciences, Islamabad (Reference: ECPIMS/19/10). Written informed consent was taken from all the study participants. The participation was on a voluntary basis. Complete anonymity was maintained throughout the research. The cases diagnosed with depression or anxiety or falling in the borderline range were referred for further psychiatric management.

Study population

All female patients, aged 18 to 40 years and diagnosed with PCOS, who presented to the Department of Gynecology during the study period were eligible to be enrolled in the study. The Rotterdam criteria was adopted for the diagnosis of PCOS [3]. Patients who were already diagnosed with depression, anxiety, or any similar mental condition were excluded from the study.

Sample size calculation

A sample size of 74 patients was calculated using OpenEpi online sample size calculator (www.OpenEpi.com) with 95% confidence level and 8% margin of error. Estimated prevalence of depression in PCOS patients of 15% was used for calculation based on the study by Batool et al. [8].

Sampling technique

Patients fulfilling the inclusion criteria and providing informed consent were enrolled in the study through the non-probability consecutive sampling technique.

Study procedure

The patients were provided a pre-designed closed-ended questionnaire that included demographic details, socio-economic status, symptomatic history, and history of comorbidities. The outcome variables including the presence and severity of anxiety and depression were assessed using the Hospital Anxiety and Depression Scale (HADS) [13].

Assessments

HADS comprises 14 items scored on a Likert scale ranging from 0 to 3. Seven items correspond to anxiety and depression each. The even number questions relate to depression, and odd number questions relate to anxiety. The scores range from 0 to 21 for both domains. A score of 7 or less was considered normal, 8-10 as borderline, and 11 or above as diagnostic for anxiety or depression [13].

Statistical analysis

The data from the questionnaires were analyzed using SPSS Version 26.0 (IBM Corp., Armonk, NY, USA). Normality of the data was assessed using the Shapiro-Wilk test. As the data were normally distributed (p <0.05), means with standard deviations were used for continuous variables. The categorical data were presented as frequencies and percentages. Pearson chi-square test was performed to find any association of depression and anxiety with demographic and socio-economic variables. Fisher’s exact test was applied where expected cell count was less than 5 in one or more cells. A p-value of <0.05 was considered statistically significant.

## Results

A total of 74 patients with PCOS were included in the study. The mean age of all the participants was 26.8 ± 5.2, and the mean body mass index (BMI) was 28.7 ± 5.4. Out of these 74 cases, 46 (62.2%) were married. Level of education was postgraduate or master’s degree in 24 (32.4%) cases, followed by undergraduate or bachelor's degree in 19 (25.7%) cases. Housewife was the most common employment status that was observed in 33 (44.6%) participants, followed by students, which comprised 24 (32.4%) cases. The most common ethnic background was Punjabi, which was present in 36 (48.6%) cases. The length of diagnosis of PCOS was more than one year in 40 (54.1%) cases, and family history of PCOS was present in 18 (24.3%) cases. The demographic and socio-economic details of the study participants are given in Table 1.

**Table 1:** Demographic details of study participants (N = 74) PCOS, polycystic ovarian syndrome

Parameter	Mean ± SD
Age (years)	26.8 ± 5.2
Body mass index (kg/m^2^)	28.7 ± 5.4
Parameter	No. of patients (%)
Marital status	
Single	28 (37.8%)
Married	46 (62.2%)
Level of education	
No formal education	2 (2.7%)
Primary	3 (4.1%)
Secondary‎/matric	10 (13.5%)
High school‎/intermediate	16 (21.6%)
Undergraduate‎/bachelor's degree	19 (25.7%)
Postgraduate‎/master’s degree	24 (32.4%)
Employment	
Part-time	5 (6.8%)
Full time	10 (13.5%)
Housewife	33 (44.6%)
Student	24 (32.4%)
Unemployed	2 (2.7%)
Ethnicity	
Punjabi	36 (48.6%)
Sindhi	1 (1.4%)
Pathan	14 (18.9%)
Kashmiri	4 (5.4%)
Gilgit Baltistani	1 (1.4%)
Urdu Speaking	12 (16.2%)
Others	6 (8.1%)
Household income	
Less than Rs. 10,000	2 (2.7%)
Between Rs. 10,000 and 25,000	15 (20.3%)
Between Rs. 25,000 and 50,000	15 (20.3%)
Between Rs. 50,000 and 100,000	19 (25.7%)
More than Rs. 100,000	23 (31.1%)
Length of PCOS diagnosis	
Less than 1 month ago	9 (12.2%)
Between 1 and 6 months ago	14 (18.9%)
Between 6 months and 1 year ago	11 (14.9%)
More than 1 year ago	40 (54.1%)
Family history of PCOS and infertility	
PCOS	18 (24.3%)
Infertility	1 (1.4%)
Both	9 (12.2%)
Neither	46 (62.2%)
Current pregnancy	
Yes	8 (10.8%)
No	66 (89.2%)
Have children	
Yes	17 (23.0%)
No	57 (77.0%)
PCOS-related symptoms	
Yes	74 (100.0%)
No	0 (0%)
Diabetes mellitus	
Yes	3 (4.1%)
No	71 (95.9%)
Hypertension	
Yes	2 (2.7%)
No	72 (97.3%)
Other comorbidities (chronic heart disease, liver disease, and renal failure)	
Yes	4 (5.4%)
No	70 (94.6%)
Family history of anxiety and depression	
Anxiety	8 (10.8%)
Depression	5 (6.8%)
Both	7 (9.5%)
Neither	54 (73.0%)

The presence of PCOS-related symptoms was assessed, and all 74 (100%) cases reported experiencing one or more of these symptoms. The most common symptom was menstrual cycle abnormalities, which was present in 57 (77.0%) cases. Weight gain was experienced by 50 (67.6%) cases and hirsutism by 41 (55.4%) cases. Table 2 shows the details of PCOS-related symptoms present in the study participants. The presence of comorbidities was also assessed. Diabetes mellitus and hypertension were present in only three (4.1%) and two (2.7%) cases, respectively. Only one patient reported a history of mental illness other than depression or anxiety. A positive family history of depression and/or anxiety was reported by 20 (27%) cases.

**Table 2:** PCOS-related symptoms in study participants (N = 74) PCOS, polycystic ovarian syndrome

Symptom		No. of patients (%)
Menstrual cycle	Regular	17 (23.0%)
	Irregular	55 (74.3%)
	Amenorrhea	2 (2.7%)
Menstrual irregularities	Present	57 (77.0%)
	Absent	17 (23.0%)
Weight gain	Present	50 (67.6%)
	Absent	24 (32.4%)
Hirsutism	Present	41 (55.4%)
	Absent	33 (44.6%)
Alopecia	Present	38 (51.4%)
	Absent	36 (48.6%)
Acne	Present	36 (48.6%)
	Absent	38 (51.4%)
Infertility	Present	15 (20.3%)
	Absent	59 (79.7%)
Acanthosis nigricans	Present	10 (13.5%)
	Absent	64 (86.5%)
Sleep apnea	Present	5 (6.8%)
	Absent	69 (93.2%)

The presence of depression and anxiety was assessed using HADS. The mean HADS score was 7 ± 3.8 for depression and 8 ± 3.7 for anxiety. Depression was diagnosed in 13 (17.6%) cases (score ≥ 11), whereas another 13 (17.6%) cases were found to be in the borderline range for depression (score: 8-10). Anxiety was diagnosed in 15 (20.3%) cases (score ≥ 11), whereas another 17 (23%) cases were found to be in the borderline range for anxiety (score: 8-10). Table 3 represents the outcomes of the study in patients with PCOS.

**Table 3:** Study outcomes in patients with PCOS (N = 74) HADS, Hospital Anxiety and Depression Scale; PCOS, polycystic ovarian syndrome

Parameter		Mean ± SD
HADS score	Depression	7 ± 3.8
	Anxiety	8 ± 3.7
Parameter		No. of patients (%)
Depression		
Present		13 (17.6%)
Absent		61 (58.6%)
Severity of depression on HADS		
Normal		48 (64.9%)
Borderline		13 (17.6%)
Abnormal		13 (17.6%)
Anxiety		
Present		15 (20.3%)
Absent		59 (79.7%)
Severity of anxiety on HADS		
Normal		42 (56.8%)
Borderline		17 (23.0%)
Abnormal		15 (20.3%)

The study also assessed the association of various demographic and socio-economic variables with the development of depression and anxiety in patients with PCOS. Depression was found to be significantly associated with BMI (p = 0.015), level of education (p = 0.033), and monthly household income (p = 0.004). However, no statistically significant association of depression was found with age, marital status, employment status, ethnicity, current pregnancy, menstrual irregularities, comorbidities such as diabetes and hypertension, and a family history of PCOS, depression, or anxiety (p < 0.05). Anxiety was found to be associated with employment status (p = 0.009) and current pregnancy (p = 0.007). However, rest of the factors such as age, BMI, marital status, educational status, monthly household income, ethnicity, menstrual irregularities, comorbidities, and a family history of PCOS, depression, or anxiety did not show a statistically significant association with anxiety (p < 0.05).

## Discussion

The results from the present study show that the age characteristics of the participants depicted a relatively younger population. The mean BMI was slightly higher in this study and falls in the range of overweight category. Although the age characteristics of the study population may depend on the selection criteria, yet a predisposition toward the younger age group is well known. PCOS is regarded as a condition of the reproductive age. A literature review of 25 studies on PCOS conducted by Bilal et al. showed that more than half of the patients with PCOS fall in the age range of 25-34 years [14]. A study from Singapore showed that BMI was significantly higher in PCOS cases as compared to controls (p < 0.001) [15]. On the contrary, a study on medical students in Pakistan showed that more than 60% of PCOS cases had a normal BMI [16]. However, the link of obesity with PCOS is already established and is believed to be driven by various genetic and metabolic mechanisms [17].

Family history of PCOS was present in 36.5% of the cases. A local study from Hyderabad, Pakistan, showed that a positive family history of PCOS was present in almost 25% of the patients [18]. The authors from the study concluded that a strong family history in first-degree relatives is linked to genetic predisposition [18]. PCOS-related symptoms were reported by all of the study participants. Menstrual irregularities, weight gain, hirsutism, and alopecia were the most common symptoms. This falls in line with another study conducted in Pakistan, which reported the most common complications in PCOS were obesity (80%) and hyperandrogenism (77.7%) [19]. The menstrual abnormalities in this syndrome are due to hyperandrogenism, in which increased androgen levels cause an imbalance in the LH/follicle-stimulating hormone levels and, hence, anovulation or oligo-ovulation ensues [2].

The primary outcome of the current study was to assess the prevalence of depression and anxiety in patients with PCOS. Depression was found in 17.6% and anxiety in 20.3% of the cases. Literature is evident on the association between these psychological disorders and PCOS. However, there is marked variation in the prevalence trends among various studies. A study from Karachi reported depression in 28.1% and an alarmingly high prevalence of anxiety of 85.9% [20]. In a study from Multan, depression and anxiety were reported in 56.9% and 61.8% of the cases, respectively [11]. Although the prevalence of these conditions may vary from population to population, it is currently believed that women with PCOS are at an eight times higher risk of developing anxiety and depression [21].

The increased incidence of depression and anxiety is believed to be due to a complex interplay of various underlying mechanisms. One widely accepted view is that the symptomatology of PCOS and these conditions overlaps and thus points toward a common association [22]. Additionally, insulin resistance, hyperandrogenism along with its related symptoms, obesity, infertility, and disturbance in the hypothalamic-pituitary-adrenal axis are some of the plausible reasons involved in the pathogenesis of these psychological conditions in patients with PCOS [23]. Having highlighted the magnitude of the mental health issues faced by PCOS patients, it is imperative to recommend the inclusion of psychiatrists in the multidisciplinary team providing treatment to these patients. Literature is evident in support of interventions for mental health, such as early screening, prompt treatment, and incorporation of various stress management strategies to decrease anxiety, depression, and stress in patients with PCOS [24].

The association of various demographic and socio-economic variables with the presence of depression and anxiety was also assessed. Depression was found to be significantly associated with BMI (p = 0.015), level of education (p = 0.033), and monthly household income (p = 0.004). Lower socio-economic status and education level were associated with higher chances of depression. A similar study from Poland also found a lower level of education to be associated with depression in women with PCOS [25]. The authors concluded that lower socio-economic status and level of education are interlinked and can determine the probability of an individual seeking timely medical attention and help in coping with stress [25]. Anxiety was found to be associated with employment status (p = 0.009) and current pregnancy (p = 0.007). Full-time employment was associated with higher chances of anxiety. Contrary to this, a Saudi study showed that unemployment was a strong predictor of anxiety in PCOS patients and maintained that unemployment results in financial insecurity and low self-esteem, both of which can lead to anxiety [26]. The relationship of prenatal anxiety with PCOS is also well-known. Koric et al. in their study on PCOS patients from Utah, USA, reported that almost one in every four pregnant females with PCOS suffer from prenatal anxiety and suggested that screening for anxiety should be offered to these patients [27].

The association of other patients’ characteristics and demographic variables, such as age, marital status, ethnicity, menstrual irregularities, comorbidities such as diabetes and hypertension, and a family history of PCOS, with either depression or anxiety was not established in this study (p < 0.05). However, literature is evident about the possible role of various associated factors in the pathogenesis of these psychological conditions such as age, clinical characteristics, comorbidities particularly insulin resistance, and even ethnicity [28,29]. Thus, the development of psychological disorders in PCOS appears to be multifactorial, which may not be fully explained by identification of a few factors. A detailed description of all these associated factors will be beyond the scope of this study. Nevertheless, the authors of the current study have tried to explore the role of certain common factors that are presumed to affect the development of depression and anxiety in women with PCOS.

Limitations

This study was limited due to it being only conducted at one hospital and having a small sample size, which may make extrapolation a challenge. The study setting involved a tertiary care hospital in a large metropolitan city. The situation in the far-flung rural areas with limited healthcare facilities may be altogether different, which demands further exploration through studies conducted in those areas. Furthermore, HADS was used to determine the level of anxiety and depression in PCOS patients instead of a psychiatric referral. A formal psychiatric evaluation by a trained psychiatrist may yield a more reliable diagnosis. The study employed quantitative methods only. Further qualitative studies can provide a deeper insight into the factors leading to depression and anxiety in patients with PCOS.

## Conclusions

Our study found that depression and anxiety are common in patients with PCOS. Various demographic and socio-economic factors such as BMI, level of education, monthly household income, employment status, and pregnancy were found to be associated with the development of psychological disorders in these patients. This indicates the need to involve a multidisciplinary team, including gynecologists and psychiatrists, to screen and manage these issues effectively.
